# An assessment of trochlear groove morphology with the severity of medial patellar luxation using ultrasonography in dogs presented to a clinic in Japan

**DOI:** 10.1002/vro2.70010

**Published:** 2025-05-14

**Authors:** Tsunenori Morishima, Koji Tanegashima, Yuma Tomo, Hinano Eto, Atsushi Yamazaki, Kazuya Edamura

**Affiliations:** ^1^ Midori Animal Care Nagoya Japan; ^2^ Laboratory of Veterinary Surgery Department of Veterinary Medicine College of Bioresource and Sciences Nihon University Fujisawa Japan

## Abstract

**Background:**

Ultrasonography is a valuable tool for the preoperative morphological evaluation of the femoral trochlear groove in dogs with medial patellar luxation (MPL). This study aimed to objectively evaluate the femoral trochlear groove morphology using ultrasonography and compare it with the severity of MPL in dogs.

**Methods:**

Small‐breed dogs weighing 10 kg or less and diagnosed with normal stifle joints or MPL were included. Pelvic limbs without any orthopaedic disease served as the normal group. Pelvic limbs with MPL were divided into grade 1, 2, 3 and 4 MPL groups according to the Singleton grading system. In this study, the heights of the medial and lateral trochlear ridges, the femoral trochlear depth and the sulcus angle (SA) were objectively measured using ultrasonography.

**Results:**

A total of 125 pelvic limbs (in 88 dogs) were evaluated: normal (*n* = 29) or grade 1 (*n* = 9), grade 2 (*n* = 42), grade 3 (*n* = 34) and grade 4 (*n* = 11) MPL groups. The heights of both the medial and lateral trochlear ridges were significantly lower in the grade 4 MPL group than in the normal and grade 1 MPL groups. The femoral trochlear depth was significantly shallower with increasing MPL grade. The SA was significantly higher in the grade 4 MPL group compared to the other groups.

**Conclusion:**

The morphology of the femoral trochlear groove can be easily and objectively evaluated using ultrasonography. Significant hypoplasia of the femoral trochlear groove was observed in the grade 4 MPL group.

## INTRODUCTION

Medial patellar luxation (MPL) is defined as the medial displacement of the patella from the femoral trochlear groove. It is one of the most common orthopaedic conditions in small animal practice.^1^, ^2^, ^3^ Medial patellar luxation is more prevalent in small‐breed dogs, including Toy Poodle, Chihuahua, Pomeranian, Yorkshire Terrier, Maltese and Papillon, and often develops at a young age.^1^, ^4^, ^5^, ^6^ Bone deformities, such as coxa vara, decrease in the anteversion angle of femoral neck, varus deformity of the distal one‐third of the femur, lateral torsion of the distal femur, shallow femoral trochlear groove with poorly developed or absent medial trochlear ridge, medial displacement of the tibial tuberosity and internal torsion of the proximal tibia, occur depending on the severity of MPL.^1^, ^4^, ^5^, ^6^, ^7^, ^8^, ^9^

Because the femoral trochlear groove is often shallow in dogs with MPL, most cases are treated with trochleoplasty as part of MPL corrective surgery. In many cases, the decision to perform trochleoplasty is based on subjective visual inspection following arthrotomy and the clinician's experience. Therefore, establishing a method to easily and objectively determine the morphology of the femoral trochlear groove, preoperatively, is desirable.

The depth of the femoral trochlear groove has traditionally been evaluated using the skyline views of the stifle joint on radiography.^10^ However, the skyline views are not consistent when evaluating the femoral trochlear groove because it depends on the angle of the stifle joint during imaging.^10^ Computed tomography (CT) has been used to evaluate the femoral trochlear groove to more accurately determine the morphology.^11^, ^12^, ^13^ While CT allows for three‐dimensional evaluation of bone morphology without being affected by the positioning of dogs during imaging, it is expensive and requires sedation or general anaesthesia.^11^

Ultrasonography is a diagnostic imaging tool for orthopaedic disease that is non‐invasive, does not require general anaesthesia and does not involve expose to radiation, making it practical for use in examination rooms. In humans, several studies have evaluated the morphology of the femoral trochlear groove using ultrasonography.^14^, ^15^, ^16^ The morphology of the femoral trochlear groove can be evaluated preoperatively using ultrasonography in dogs. However, to the best of the authors’ knowledge, there has been one report about the ultrasonographic evaluation of the morphology of the femoral trochlear groove in dogs,^17^ with no studies comparing the severity of grades 1‒4. We hypothesised that ultrasonography might be a useful imaging tool that could easily demonstrate the morphology of the femoral trochlear groove preoperatively in dogs with MPL. Our study aimed to objectively evaluate the morphology of the femoral trochlear groove using ultrasonography and compare it with the severity of MPL in dogs.

## METHODS

### Case selection

This study included small‐breed dogs weighing 10 kg or less that presented to the Midori Animal Care (MAC) clinic between February and May 2016 and were diagnosed with MPL using palpation. Pelvic limbs with MPL were divided into grade 1, 2, 3 and 4 MPL groups according to the Singleton grading system.^18^ Dogs with other orthopaedic diseases affecting the pelvic limbs were excluded from the study. Pelvic limbs without any orthopaedic diseases, including MPL using palpation and radiography, were included as the normal group. This study was conducted with the approval of the director of the MAC clinic (MAC020115), and all dog owners gave written consent for data collection for this study.

### Ultrasonography

**FIGURE 1:**
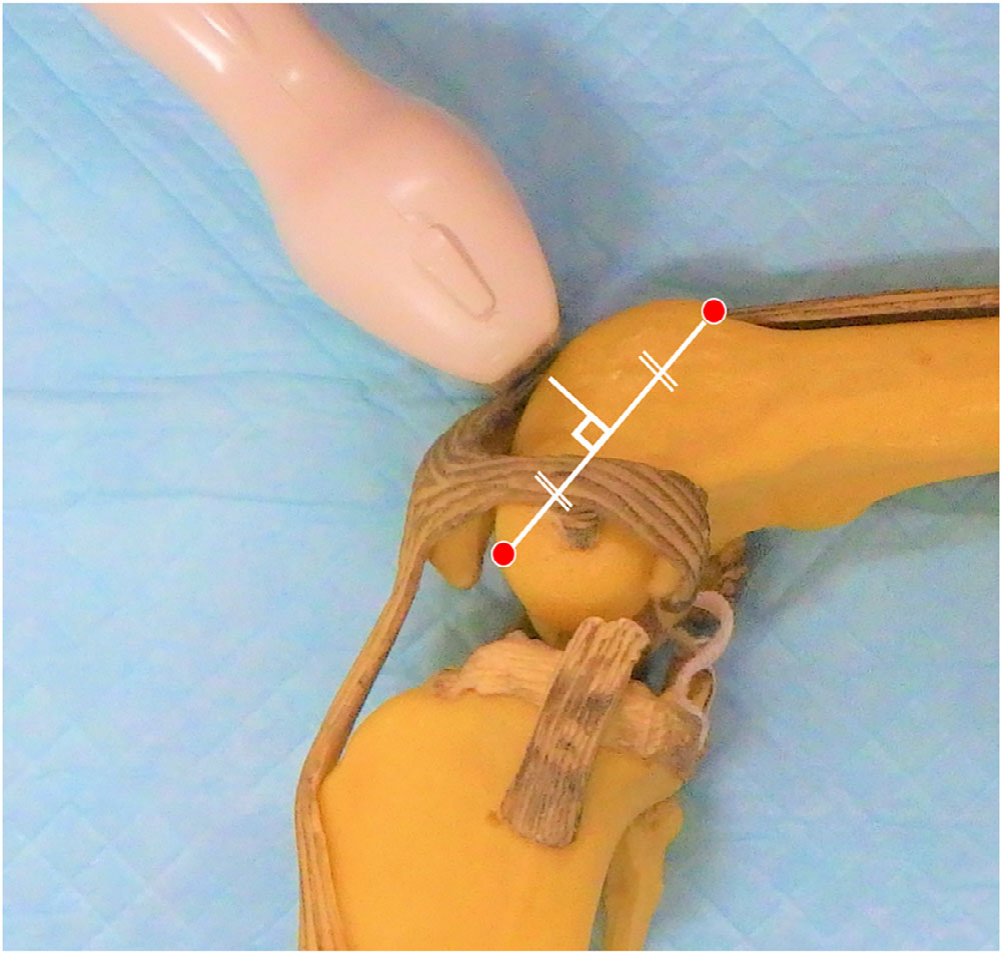
Application of probe to the femoral trochlear groove in the canine bone model. The dogs were placed in lateral recumbency with the stifle joint flexed at 90°. The linear probe was placed perpendicular to the midpoint of the line connecting the most distal and proximal parts of the femoral trochlear groove. The red dots indicate the most proximal and distal sites of the femoral trochlear groove.

Ultrasonographic diagnostic equipment (Aplio^TM^300 TUS‐A300, Canon Medical Systems) and 12 MHz linear array probe (PLT‐1204SBT, Canon Medical Systems) were used to evaluate the morphology of the femoral trochlear groove. The dogs were positioned in lateral recumbency, with the stifle joint flexed at 90°. No sedatives or general anaesthesia were used. Ultrasonography was performed without shaving the hair in all cases. The hair was wetted with alcohol spray, brushed off and the linear array probe placed perpendicular to the bony surface of the distal femur, just proximal to the patella, to visualise the femoral trochlear groove. This method enabled the probe to be positioned perpendicular to the midpoint of the line connecting the most distal and proximal parts of the femoral trochlear groove (Figure 1).

### Objective measurements of the femoral trochlear groove

To evaluate the morphology of the femoral trochlear groove by ultrasonography, the height of the medial and lateral trochlear ridges, depth of the femoral trochlear groove and sulcus angle (SA) were objectively measured using the following methods.

**FIGURE 2:**
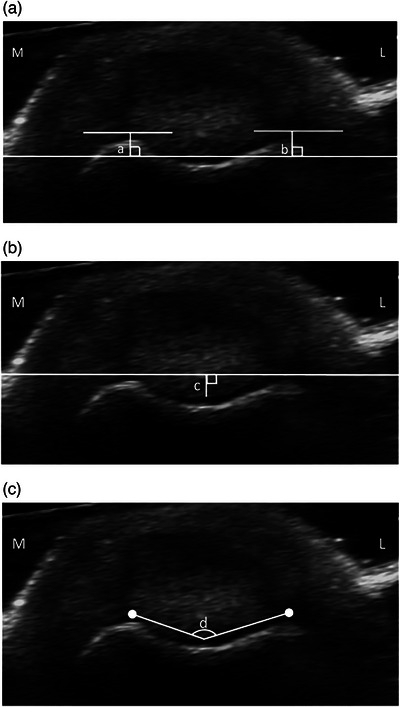
Measuring methods for the femoral trochlear groove using ultrasonography. (a) Height of the medial (a) and lateral (b) trochlear ridges. (b) Depth of the femoral trochlear groove (c). (c) Sulcus angle (SA) (d). L: lateral; M: medial.

First, the probe was placed distal to the aforementioned measurement position; the medial and lateral femoral condyles were displayed and a tangent to them was drawn. Subsequently, the probe was placed at the aforementioned measurement position and a reference line parallel to the tangent and passing through the deepest cartilaginous surface of the femoral trochlear groove was draw, followed by a vertical line passing through the highest cartilaginous point of the medial or lateral trochlear ridge. The height of the medial or lateral trochlear ridge was evaluated by measuring the distance between the highest cartilaginous point of the medial or lateral trochlear ridge and the reference line (Figure 2a, a and b).

Second, a line was drawn connecting the highest cartilaginous points of the medial and lateral trochlear ridges, followed by a perpendicular line passing through the deepest cartilaginous surface of the femoral trochlear groove; based on a previous study in humans using MRI.^19^ The depth of the femoral trochlear groove was evaluated by measuring the distance between the deepest cartilaginous surface of the femoral trochlear groove and the line passing through the highest cartilaginous points of the medial and lateral trochlear ridges (Figure 2b, c).

Finally, the SA was measured as the angle formed by the highest cartilaginous points of the medial and lateral trochlear ridges and the deepest cartilaginous point of the trochlear groove, which was based in part on another MRI study in humans (Figure 2c, d).^20^

### Statistical analysis

The measurement results are expressed as the means and standard deviations. Statistical analyses were performed using data analysis software (SPSS ver. 20; IBM Corp.). One‐way analysis of variance was used to compare groups for measurement items of the femoral trochlear groove. Tukey‒Kramer's multiple comparison test was used as the post hoc test. A *p*‐value of less than 0.05 was considered statistically significant. The correlation between bodyweight and each measurement value was evaluated by Pearson's correlation analysis.

## RESULTS

A total of 125 stifle joints (in 88 dogs) were evaluated during the study period. The normal group consisted of 29 stifle joints in 15 dogs, which included three intact male, four neutered male, one intact female and seven neutered female dogs. The breeds represented were Toy Poodle (*n*  =  6), Chihuahua (*n*  =  4), Shih Tzu (*n*  =  2), miniature Dachshund (*n*  =  1), Maltese (*n*  =  1) and Yorkshire Terrier (*n*  =  1). The median age and bodyweight of the dogs were 84 months (range: 16‒163 months) and 3.65 kg (range: 2.45‒6.64 kg), respectively.

The grade 1 MPL group comprised nine stifle joints in eight dogs, which included one intact male, four neutered males, one intact female and two neutered female dogs. The breeds represented were Toy Poodle (*n*  =  4), Chihuahua (*n*  =  2), Shih Tzu (*n*  =  1) and miniature Dachshund (*n*  =  1). The median age and bodyweight of the dogs were 69 months (range: 16‒156 months) and 4.40 kg (range: 3.35‒9.20 kg), respectively.

The grade 2 MPL group comprised 42 stifle joints in 33 dogs. This group included five intact males, five neutered males, seven intact females and 16 neutered females. The breeds represented in this group were Toy Poodle (*n*  =  9), Chihuahua (*n*  =  7), Shih Tzu (*n*  =  5), Yorkshire Terrier (*n*  =  3), Pomeranian (*n*  =  3), Maltese (*n* = 1), Petit Brabancon (*n*  =  1) and mixed breed (*n*  =  4). The median age and bodyweight of the dogs were 60 months (range: 8‒163 months) and 3.20 kg (range: 1.72‒9.20 kg), respectively.

The grade 3 MPL group comprised 34 stifle joints in 25 dogs, which included two intact males, three neutered males, six intact females and 14 neutered females. The breeds represented were Chihuahua (*n*  =  13), Toy Poodle (*n*  =  5), Yorkshire Terrier (*n*  =  2), miniature Dachshund (*n*  =  1), Pomeranian (*n*  =  1) and mixed breed (*n*  =  3). The median age and bodyweight of the dogs were 64 months (range: 8‒144 months) and 3.20 kg (range: 1.78‒7.65 kg), respectively.

The grade 4 MPL group consisted of 11 stifle joints in seven dogs. This group included one intact male, two neutered males, two intact females and two neutered females. The breeds represented in this group were Chihuahua (*n*  =  2), Yorkshire Terrier (*n*  =  2), Toy Poodle (*n*  =  1), Petit Brabancon (*n*  =  1) and mixed breed (*n*  =  1). The median age and bodyweight of the dogs were 72 months (range: 9‒132 months) and 2.3 kg (range: 1.7‒3.9 kg), respectively. No significant differences were observed between the groups for sex, age and bodyweight.

**FIGURE 3:**
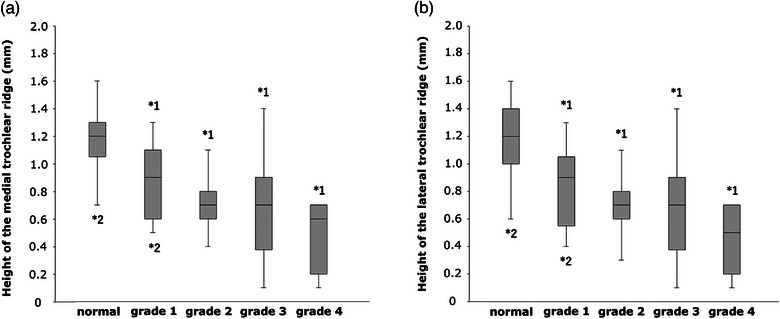
(a and b) Box plots showing the height of the medial and lateral trochlear ridges. *1 indicates a significant difference from the normal group (*p* < 0.05). *2 indicates a significant difference from the grade 4 medial patellar luxation group (*p* < 0.05).

The heights of the medial trochlear ridge in the normal, grade 1, 2, 3 and 4 MPL groups were 1.16 ± 0.20 mm, 0.89 ± 0.26 mm, 0.69 ± 0.18 mm, 0.67 ± 0.31 mm and 0.46 ± 0.23 mm, respectively. The height of the medial trochlear ridge was significantly greater in the normal group than in all MPL groups (grade 1 MPL group: *p* = 0.03; grades 2, 3 and 4 MPL groups: *p* < 0.001) (Figure 3a). The height of the medial trochlear ridge was significantly lower in the grade 4 MPL group than in the normal (*p* < 0.001) and grade 1 MPL groups (*p* = 0.001) (Figure 3a).

The heights of the lateral trochlear ridge in the normal, grade 1, 2, 3 and 4 MPL groups were 1.16 ± 0.25 mm, 0.81 ± 0.29 mm, 0.70 ± 0.17 mm, 0.67 ± 0.32 mm and 0.47 ± 0.22 mm, respectively. The height of the lateral trochlear ridge was significantly greater in the normal group than in all MPL groups (grade 1 MPL group: *p* = 0.004; grade 2, 3 and 4 MPL groups: *p* < 0.001) (Figure 3b). The height of the lateral trochlear ridge was significantly lower in the grade 4 MPL group than in the normal (*p* < 0.001) and grade 1 MPL groups (*p* = 0.03) (Figure 3b).

**FIGURE 4:**
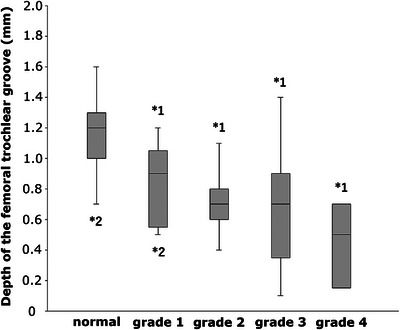
Box plots showing the depth of the femoral trochlear groove. *1 indicates a significant difference from the normal group (*p* < 0.05). *2 indicates a significant difference from the grade 4 medial patellar luxation group (*p* < 0.05).

The depths of the femoral trochlear groove in the normal, grade 1, 2, 3 and 4 MPL groups were 1.17 ± 0.21 mm, 0.83 ± 0.25 mm, 0.69 ± 0.17 mm, 0.66 ± 0.31 mm and 0.46 ± 0.22 mm, respectively. The depth of the femoral trochlear groove was significantly deeper in the normal group than in all MPL groups (grade 1 MPL group: *p* = 0.003; grade 2, 3 and 4 MPL groups: *p* < 0.001). The depth of the femoral trochlear groove was significantly shallower in the grade 4 MPL group than in the normal (*p* < 0.001) and grade 1 MPL groups (*p* = 0.008) (Figure 4).

**FIGURE 5:**
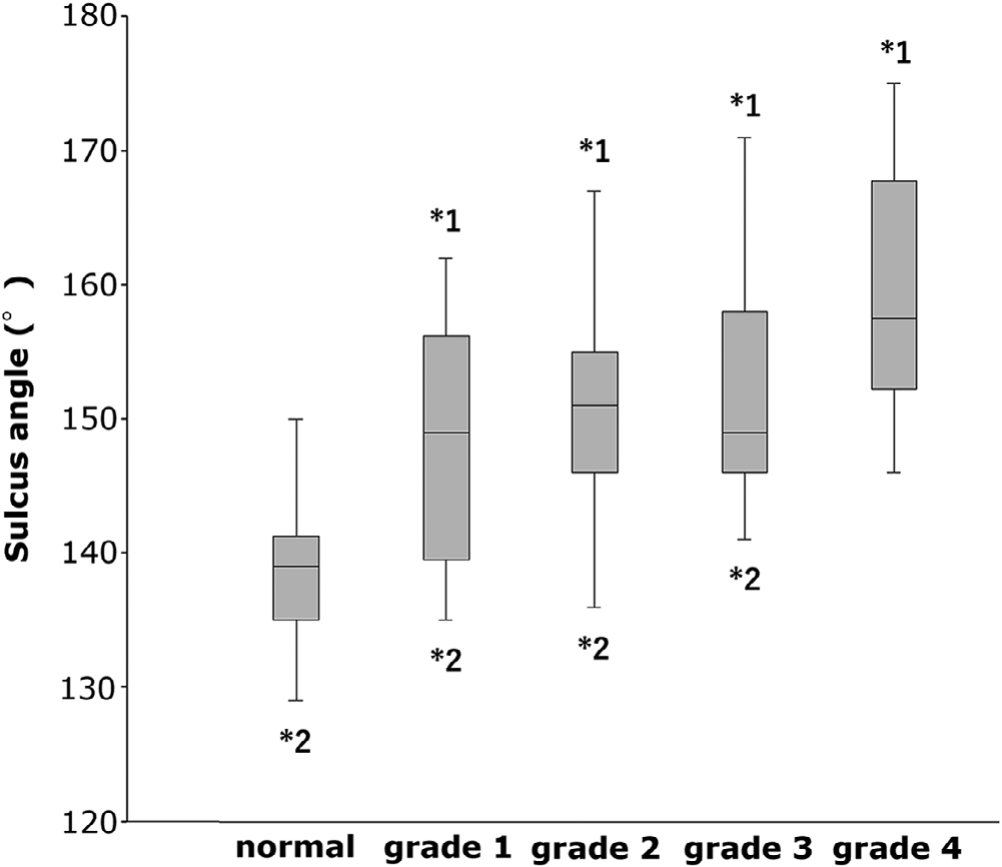
Box plots showing data for the sulcus angle. *1 indicates a significant difference from the normal group (*p* < 0.05). *2 indicates a significant difference from the grade 4 medial patellar luxation group (*p* < 0.05).

**FIGURE 6:**
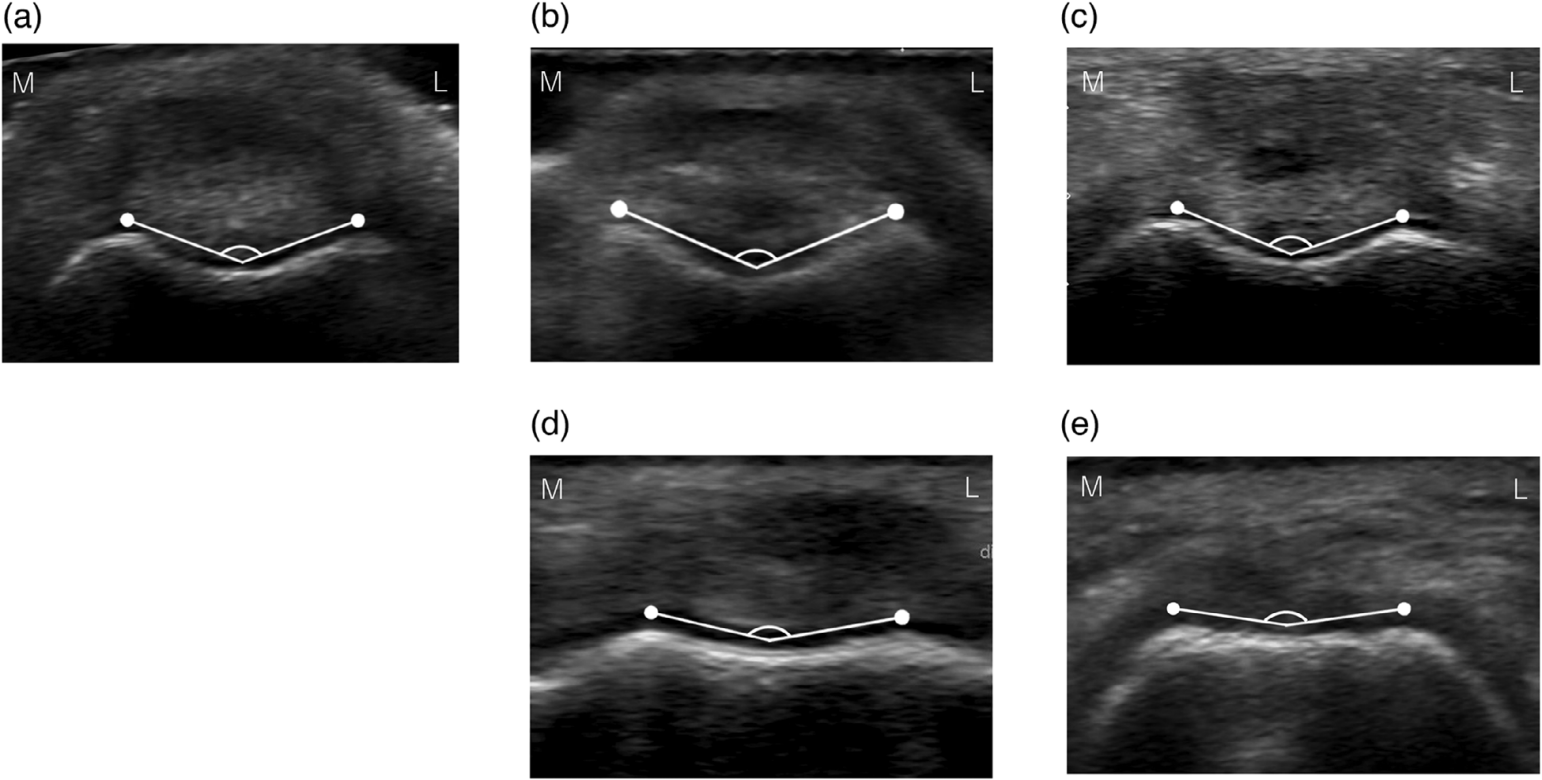
Ultrasonographic findings of the trochlear groove. Angle shown in the figure is the sulcus angle (SA)—this tended to become higher and the trochlear groove shallower with increasing medial patellar luxation (MPL) grade. (a) Normal, (b) grade 1 MPL, (c) grade 2 MPL, (d) grade 3 MPL and (e) grade 4 MPL. L: lateral; M: medial.

The SA values in the normal, grade 1, 2, 3 and 4 MPL groups were 139.00 ± 4.59°, 150.00 ± 7.90°, 151.31 ± 7.03°, 152.74 ± 8.20° and 159.82 ± 8.65°, respectively. The SA was significantly smaller in the normal group than in all MPL groups (grade 1 MPL group: *p* = 0.001; grade 2, 3 and 4 MPL groups: *p* < 0.001). The SA was significantly greater in the grade 4 MPL group than in the other groups (normal group: *p* < 0.001; grade 1 MPL group: *p* = 0.03; grade 2 MPL group: *p* = 0.006; grade 3 MPL group: *p* = 0.04) (Figure 5). Thus, SA was higher as the grade of MPL increased (Figure 6).

**FIGURE 7:**
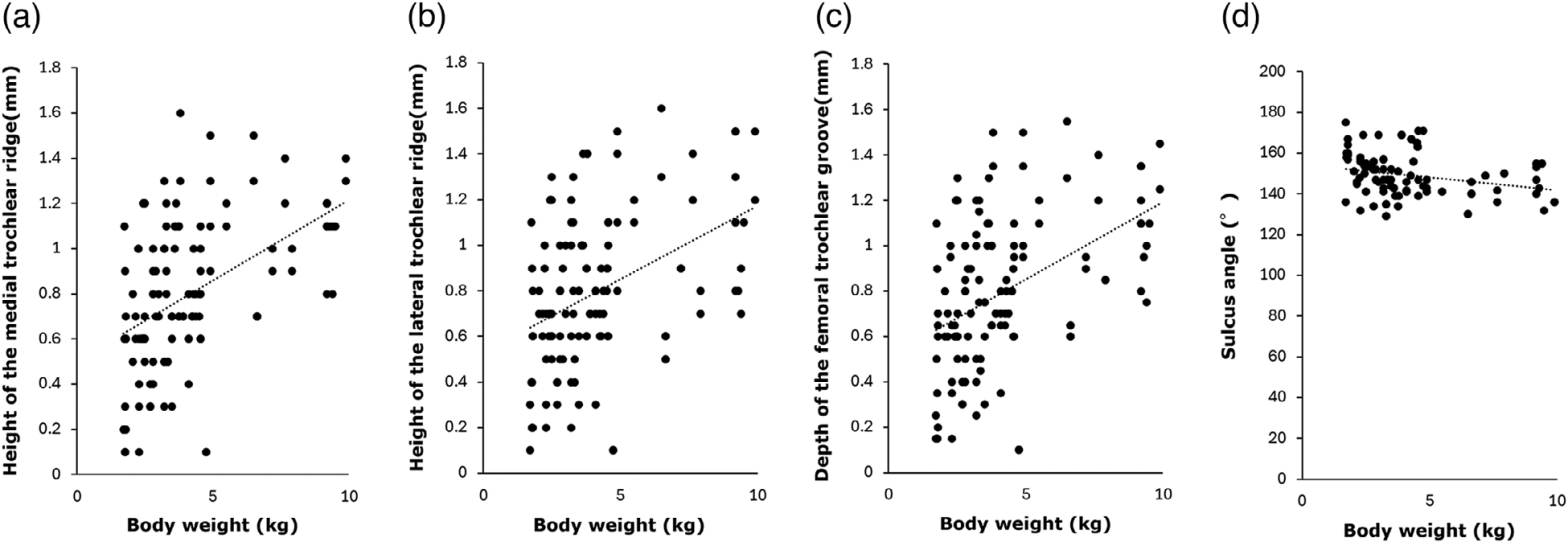
Correlation between bodyweight and measured values. The heights of the medial and lateral trochlear ridges and the depth of the femoral trochlear groove tended to be greater as bodyweight increased. While the sulcus angle was not affected by bodyweight, variation in measurement values was minimal. (a) Height of the medial trochlear ridge, (b) height of the lateral trochlear ridge, (c) depth of the femoral trochlear groove and (d) sulcus angle.

The correlation between bodyweight and each of the measurement values showed positive correlations for the heights of the medial (*r* = 0.47, *p* < 0.001) and lateral trochlear ridges (*r* = 0.42, *p* < 0.001) and the depth of the femoral trochlear groove (*r* = 0.45, *p* < 0.001). Thus, the heights of the medial and lateral trochlear ridges and the depth of the femoral trochlear groove correlated to be greater as bodyweight increased. On the other hand, SA was not affected by bodyweight increase (*r* = ‒0.27, *p* = 0.003) and had the smallest variation in measurements (Figure 7).

## DISCUSSION

In the present study, the morphology of the femoral trochlear groove was easily visualised using ultrasonography in all dogs without anaesthesia or radiation exposure. This study demonstrated that dogs with MPL tended to have lower medial and lateral trochlear ridge heights, shallower femoral trochlear grooves and higher SA values than dogs without MPL. In addition, the heights of the medial and lateral trochlear ridges and the depth of the femoral trochlear groove tended to decrease with increasing MPL severity.

The evaluation of the trochlear groove morphology has been mainly performed using CT in dogs.^11^, ^21^, ^22^ However, since CT does not visualise the articular cartilage, ultrasonography is more useful for accurately evaluating the morphology of the femoral trochlear groove. In humans, there has been a report on measuring the SA at the bony and cartilaginous surfaces of the femoral trochlear groove using ultrasonography, and the changes in the morphology with growth were compared.^15^ In dogs, a similar study has been reported in which the femoral trochlear groove was evaluated using ultrasonography at the bony and cartilaginous surfaces, and the changes in the morphology with growth have been compared.^17^ Additionally, bony SA changed with growth up to 6 months of age, while cartilaginous SA (CSA) rarely changed.^17^ In addition, the CSA measured using ultrasonography correlated with the CSA measured using magnetic resonance imaging, indicating that ultrasonography could accurately determine the morphology of the femoral trochlear groove in dogs.^17^ Therefore, in the present study, the morphology of the femoral trochlear groove was evaluated using the cartilaginous surface in dogs aged 8 months or older. The previously reported CSA measured using ultrasonography in dogs under 1 and 3 years of age were 139.0 ± 5.4° and 139.4 ± 6.5°, respectively.^17^ In the present study, the SA in the normal group was 139.00 ± 4.59°, similar to previous reports. There were two reports that compared SA in normal and MPL‐affected dogs using radiography.^23^, ^24^ In those reports, SA was larger in MPL‐affected dogs than in healthy dogs, and SA was larger in dogs with higher grade of MPL. The results of the present study showed a similar trend to these studies.

To the best of the authors’ knowledge, there have been no reports on evaluating the height of the trochlear ridge using ultrasonography in dogs, although there has been a report on measuring the depth of the femoral trochlear groove.^25^ A study involving dogs weighing 5‒36 kg affected with patellar luxation, which measured the depth of the femoral trochlear groove using ultrasonography, reported that the depth was 1.5 ± 0.43 mm.^25^ It has also been reported that the depth of the trochlear groove could be accurately measured using ultrasonography because the actual depth of the trochlear groove measured during surgery was 1.5 ± 0.49 mm.^25^ In the present study, the depth of the trochlear groove in the normal group was 1.17 ± 0.21 mm, which is smaller than previously reported. This is potentially because this study was performed with dogs weighing less than 10 kg, whereas, to the best of the authors’ knowledge, previous studies involved larger dogs.

To the best of the authors’ knowledge, no published studies have objectively compared the morphology of the femoral trochlear groove among all grades of MPL using ultrasonography. This study is the first to objectively demonstrate that the femoral trochlear groove becomes shallower as the MPL grade increases using ultrasonography. In the present study, all measured parameters in the MPL group were significantly different from those in the normal group. Previous report has described that the morphology of the femoral trochlear groove does not change until grade 2 MPL.^12^ However, there is a report indicating that the morphology of the femoral trochlear groove is significantly deformed even at grade 2 MPL and our results were consistent with that report.^11^

Among the measured parameters, the SA may be the most useful for a simplified assessment of the shape of the femoral trochlear groove because the heights of trochlear ridges and depth of the femoral trochlear groove depend on the size of the dog. In humans, the SA has been used to evaluate the morphology of the femoral trochlear groove in patients with patellar instability.^26^ Therefore, the SA may be the most appropriate measurement parameter for evaluating the shallowness of the femoral trochlear groove in dogs with MPL. All groups with an SA significantly higher than that of the normal group had a mean SA greater than approximately 150°. Therefore, if the SA exceeds 150°, trochleoplasty may be indicated. Thus, the measurement method used in the present study may contribute to the establishment of indications for trochleoplasty in dogs with MPL.

This study had several limitations. There was a breed bias; most of the included dogs were Toy Poodle and Chihuahua. In addition, this study was designed to include dogs weighing less than 10 kg, but it was not a single‐breed study. Therefore, differences in bone morphology between breeds could not be eliminated. Since the number of cases in the grade 1 and 4 MPL groups was small compared with that in the other groups, further investigation with a larger number of cases is required. Furthermore, only one location of the femoral trochlear groove was evaluated. As ultrasonography can easily screen the surface of the trochlear groove, further studies should be performed to determine the optimal measurement sites. The present study did not include dogs with severe varus deformity of the distal femur. In such cases, the methods of the present study may not be able to accurately evaluate the morphology of the femoral trochlear groove. In addition, further study including simultaneous measurement of patellar is needed to employ SA as an indicator for trochleoplasty.

In conclusion, the morphology of the femoral trochlear groove could be easily and objectively evaluated using ultrasonography in all subject dogs. Significant hypoplasia of the femoral trochlear groove was observed in the grade 4 MPL group. Because it is difficult to accurately determine the depth of the femoral trochlear groove by radiography, ultrasonography may be useful as a diagnostic tool to determine the morphology of the femoral trochlear groove.

## AUTHOR CONTRIBUTIONS

Tsunenori Morishima and Kazuya Edamura conceptualised and designed this study. Tsunenori Morishima analysed the survey data and wrote the initial manuscript. Tsunenori Morishima, Koji Tanegashima, Yuma Tomo, Hinano Eto, Atsushi Yamazaki and Kazuya Edamura reviewed and refined the final manuscript.

## CONFLICTS OF INTEREST

The authors declare they have no conflicts of interest.

## FUNDING INFORMATION

The authors received no specific funding for this study.

## ETHICS STATEMENT

The authors confirm that the ethical policies of the journal, as noted on the journal's author guidelines page, have been adhered to. This study was conducted with the approval of the director of the hospital and all dog owners provided written consent for data collection for this study.

## Data Availability

The data that support the findings of this study are openly available.

## References

[1] Alam MR , Lee JI , Kang HS , Kim IS , Park SY , Lee KC et al. Frequency and distribution of patellar luxation in dogs. 134 cases (2000 to 2005). Vet Comp Orthop Traumatol. 2007;20:59‒64. 17364098

[2] Hayes AG , Boudrieau RJ , Hungerford LL . Frequency and distribution of medial and lateral patellar luxation in dogs: 124 cases (1982‒1992). J Am Vet Med Assoc. 1994;205:716‒720. 7989241

[3] Roush JK . Canine patellar luxation. Vet Clin North Am Small Anim Pract. 1993;23:855‒868. 10.1016/s0195-5616(93)50087-6 8337795

[4] Piermattei DL , Flo GL . The stifle joint. In: Brinker WO , Piermattei DL , Flo GL , editors. Handbook of small animal orthopedics and fracture repair. 4th ed. Philadelphia, PA, USA: Saunders; 2006. p. 562‒582.

[5] Priester WA . Sex, size, and breed as risk factors in canine patellar luxation. J Am Vet Med Assoc. 1972;160:740‒742. 5010616

[6] LaFond E , Breur GJ , Austin CC. Breed susceptibility for developmental orthopedic diseases in dogs. J Am Anim Hosp Assoc. 2002;38:467‒477. 10.5326/0380467 12220032

[7] Swiderski JK , Palmer RH . Long‐term outcome of distal femoral osteotomy for treatment of combined distal femoral varus and medial patellar luxation: 12 cases (1999‒2004). J Am Vet Med Assoc. 2007;231:1070‒1075. 10.2460/javma.231.7.1070 17916032

[8] Hulse DA . Pathophysiology and management of medial patellar luxation in the dog. Vet Med Small Anim Clin. 1981;76:43‒51. 6906917

[9] Mortari AC , Rahal SC , Vulcano LC , Silva VC , Volpi RS . Use of radiographic measurements in the evaluation of dogs with medial patellar luxation. Can Vet J. 2009;50:1064‒1068. 20046606 PMC2748288

[10] Ferguson J . Patellar luxation in the dog and cat. In Pract. 1997;19:174‒184. 10.1136/inpract.19.4.174

[11] Yasukawa S , Edamura K , Tanegashima K . Morphological analysis of bone deformities of the distal femur in toy poodles with medial patellar luxation. Vet Comp Orthop Traumatol. 2021;34:303‒311. 10.1055/s-0041-1726084 33979880

[12] Sasaki A , Hidaka Y , Mochizuki M . Computed tomographic measurements of the sulcus angle of the femoral trochlea in small‐breed dogs with and without medial patellar luxation. Vet Comp Orthop Traumatol. 2022;35:314‒320. 10.1055/s-0042-1749151 35760367

[13] Nicetto T , Longo F , Contiero B , Isola M , Petazzoni M . Computed tomographic localization of the deepest portion of the femoral trochlear groove in healthy dogs. Vet Surg. 2020;49:1246‒1254. 10.1111/vsu.13426 32343440

[14] Martino F , De Serio A , Macarini L , Colaianni P , Solarino M , Fracella MR . The sulcus angle of the femoral trochlea: ultrasonographic evaluation. Radiol Med. 1995;89:215‒218. [Article in Italian] 7754110

[15] Nietosvaara Y . The femoral sulcus in children. An ultrasonographic study. J Bone Joint Surg Br. 1994;76:807‒809. 8083274

[16] Toms AP , Cahir J , Swift L , Donell ST . Imaging the femoral sulcus with ultrasound, CT, and MRI: reliability and generalizability in patients with patellar instability. Skeletal Radiol. 2009;38:329‒338. 10.1007/s00256-008-0639-9 19183987

[17] Sasaki A , Hidaka Y , Mochizuki M , Honnami M . Measurement of femoral trochlear morphology in dogs using ultrasonography. Vet Comp Orthop Traumatol. 2023;36:294‒301. 10.1055/s-0043-1770902 37487535

[18] Singleton WB . The surgical correction of stifle deformities in the dog. J Small Anim Pract. 1969;10:59‒69. 10.1111/j.1748-5827.1969.tb04021.x 5812819

[19] Pfirrmann CW , Zanetti M , Romero J , Hodler J . Femoral trochlear dysplasia: MR findings. Radiology. 2000;216:858‒864. 10.1148/radiology.216.3.r00se38858 10966723

[20] Kalichman L , Zhu Y , Zhang Y , Niu J , Gale D , Felson DT , et al. The association between patella alignment and knee pain and function: an MRI study in persons with symptomatic knee osteoarthritis. Osteoarthritis Cartilage. 2007;15:1235‒1240. 10.1016/j.joca.2007.04.014 17570690

[21] Towle HA , Griffon DJ , Thomas MW , Siegel AM , Dunning D , Johnson A . Pre‐ and postoperative radiographic and computed tomographic evaluation of dogs with medial patellar luxation. Vet Surg. 2005;34:265‒272. 10.1111/j.1532-950x.2005.00040.x 16115084

[22] Longo F , Memarian P , Christoph S , Contiero B , Pozzi A . Computed tomographic measurements of the femoral trochlea in dogs with and without medial patellar luxation. Vet Surg. 2023;52:395‒406. 10.1111/vsu.13903 36196803

[23] Garnoeva R . Evaluation of trochlear dysplasia in dogs with medial patellar luxation—comparative studies. Acta Sci Vet. 2021;49:1845. 10.22456/1679-9216.118579

[24] Garnoeva RS . Diagnostic value of patellofemoral parameters in small breed dogs with medial patellar luxation: a tangential X‐ray study. Austral J Vet Sci. 2023;55:189‒196. 10.4206/ajvs.553.05

[25] Hansen JS , Lindeblad K , Buelund L , Miles J . Predicting the need for trochleoplasty in canine patellar luxation using pre‐ and intra‐operative assessments of trochlear depth. Vet Comp Orthop Traumatol. 2017;30:131‒136. 10.3415/VCOT-16-06-0084 28094422

[26] Dejour D , Le Coultre B . Osteotomies in patello‐femoral instabilities. Sports Med Arthrosc Rev. 2007;15:39‒46. 10.1097/JSA.0b013e31803035ae 17301701

